# Electrochemical Performance of Guanidinium Salt-Added PVP/PEO Solid Polymer Electrolyte with Superior Power Density

**DOI:** 10.3390/polym17020206

**Published:** 2025-01-15

**Authors:** Anbazhagan Murugan, Vadivel Siva, Abdul Samad Shameem, Paranthaman Vijayakumar, Arangarajan Viji, Jintae Lee, Govindasamy Palanisamy

**Affiliations:** 1 Department of Science and Humanities, Karpagam College of Engineering, Coimbatore 641032, Tamil Nadu, India; 2 Department of Physics, Centre for Energy and Environment, Karpagam Academy of Higher Education, Coimbatore 641021, Tamil Nadu, India; 3 Department of Science and Humanities, Karpagam Academy of Higher Education, Coimbatore 641021, Tamil Nadu, India; 4 Department of Electrochemistry, Saveetha School of Engineering, SIMATS, Chennai 602105, Tamil Nadu, India; vijayakumar@uniten.edu.my; 5 Department of Physics, Kongunadu College of Engineering and Technology, Thottiyam, Tiruchirappalli 621215, Tamil Nadu, India; 6 School of Chemical Engineering, Yeungnam University, 280 Daehak-ro, Gyeongsan 38541, Republic of Korea

**Keywords:** solid polymer electrolyte, poly(vinyl pyrrolidone), polyethylene oxide, dielectric, supercapacitors

## Abstract

Solid polymer electrolytes (SPEs) for symmetrical supercapacitors are proposed herein with activated carbon as electrodes and optimized solid polymer electrolyte membranes, which serve as the separators and electrolytes. We propose the design of a low-cost solid polymer electrolyte consisting of guanidinium nitrate (GuN) and poly(ethylene oxide) (PEO) with poly(vinylpyrrolidone) (PVP). Using the solution casting approach, blended polymer electrolytes with varying GuN weight percentage ratios of PVP and PEO are prepared. On the blended polymer electrolytes, structural, morphological, vibrational, and ionic conductivity are investigated. The solid polymer electrolytes’ morphology and level of roughness are examined using an FESEM. The interlinking bond formation between the blended polymers and the GuN salt is verified by FTIR measurements, indicating that the ligands are chemically complex. We found that, up to 20 wt.% GuN, the conductivity value increased (1.84 × 10^−6^ S/cm) with an increase in mobile charge carriers. Notably, the optimized PVP/PEO/20 wt.% solid polymer electrolyte was fabricated into a solid-state symmetrical supercapacitor device, which delivered a potential window of 0 to 2 V, a superior energy density of 3.88 Wh kg^−1^, and a power density of 1132 W kg^−1^.

## 1. Introduction

For the sustainable growth of humanity, supercapacitors (SCs) are among the most effective energy storage technologies to address the major problems of environmental pollution and energy scarcity. The material of the electrodes is crucial to the electrochemical performance of a supercapacitor, which is made up of two electrodes: an electrolyte and a separator [1,2,3,4]. A possible strategy to improve the performance of supercapacitors is the preparation of solid polymer electrolytes, especially mixes of polyvinylpyrrolidone (PVP) and polyethylene oxide (PEO). PVP is well known for its exceptional solubility and ability to form films, whereas PEO, when complexed with lithium ions, significantly increases ionic conductivity [5,6,7]. When combined, these polymers can provide a flexible electrolyte matrix that facilitates effective ion transport, which is essential for supercapacitors’ quick cycles of charging and discharging. This combination increases mechanical stability in addition to ionic conductivity, which is crucial for preserving the supercapacitor’s structural integrity while it is operating [8].

Adding PVP and PEO to supercapacitor designs has a number of benefits, such as lighter weight and enhanced safety. Solid polymer electrolytes improve device reliability by reducing the dangers of liquid electrolytes, such as flammability and leaking [9]. Additionally, because these polymer blends are lightweight, flexible supercapacitor devices that are appropriate for wearable electronics and portable energy storage systems can be developed. Furthermore, customized electrochemical interfaces that maximize charge transfer and energy storage capacities are made possible by PVP and PEO’s compatibility with a range of electrode materials [10,11]. Optimizing the ionic conductivity of PVP/PEO solid polymer electrolytes at room temperature is still difficult despite their benefits. In addition to investigating the impacts of various processing methods, research is concentrated on improving the ion transport capabilities by adding different salts and plasticizers [12,13]. Furthermore, long-term stability and performance depend on an understanding of these polymers’ mechanical behavior throughout charge–discharge cycles. The efficiency and scalability of supercapacitor technologies could be greatly increased with further developments in this field, opening the door for their incorporation into next-generation energy storage systems [14]. A novel polymeric material with a wide range of potential applications have been developed and designed using the polymer blending process. Two major benefits of polymer blending are the simplicity of synthesis conditions and the appropriate control of physical properties through compositional changes [15,16]. Poly (ethylene oxide) (PEO)-based electrolytes have been extensively researched and utilized as key constituents in solid polymer electrolytes due to their favorable mechanical, thermal, and interfacial stability when combined with lithium metal [17,18]. PEO has the ability to form hydrogen bonds between molecules with a variety of species that are very electronegative. Additionally, the PVP polymer contains ternary amide and electronegative oxygen groups, which are possible exceptional proton acceptors [12,16]. PEO and PVP must interact with one another through inter-chain hydrogen bonding when these two polymers are combined.

Guanidinium salts are compatible with non-aqueous solvents such organic carbonates found in lithium-ion batteries. This compatibility improves the overall performance of the electrolyte in systems that use these solvents. Guanidinium nitrate is a salt that improves the characteristics of polymer electrolytes in a variety of applications, including solid-state batteries and other electrochemical devices [12]. PEO’s structure, which includes ether–oxygen bonds at advantageous interatomic distances, makes it easier for the polymeric chain to move in segments, which in turn aids in easy ionic conduction. The crystalline and amorphous phases that make up PEO’s microstructure have a significant impact on the ion transport characteristics [11,19]. By adding additional active elements and modifying the final polymer structure, PEO-based systems’ electrochemical characteristics can be greatly enhanced. When creating a polymer blend with PEO, PVP is chosen as the second polymer due to certain special characteristics. When compared to similar polymers, PVP has several advantageous qualities, including rich physics in the charge transport mechanism, good environmental stability, and moderate electrical conductivity [12,20]. This present study involves a hydrogen-rich guanidinium salt-added PVP/PEO system and its dielectric and electrochemical properties.

## 2. Experimental Details

### 2.1. Materials

AR-grade chemicals were purchased and used without further purification. Guanidinium nitrate (Merck, Rahway, NJ, USA), poly(vinyl pyrrolidone) (Sigma Aldrich, St. Louis, MO, USA), polyethylene oxide (Merck), and double-distilled (DD) water were used for the preparation of solid polymer electrolytes.

### 2.2. Preparation of PVP/PEO/Guanidium Nitrate Electrolytes

Firstly, 70 wt.% PVP, 30 wt.% PEO, and x wt.% of guanidium nitrate (x = 5, 10, 15, 20, 25)-based polymer electrolytes were prepared using the solution casting method. The PVP was added to distilled water to obtain a clear transparent solution. Likewise, PEO and guanidium nitrate solution were prepared separately. To prepare a homogenous solutions were combined and vigorously stirred for a full day. To ensure that any last solvent traces had been eliminated, the resultant solution was transferred onto polypropylene plates and allowed to dry at room temperature. Uniform and flexible films were acquired and kept in desiccators for further analysis.

### 2.3. Electrochemical Studies and Device Fabrication

The solid-state symmetric supercapacitor was fabricated using the PVP/PEO/20 wt.% GuN solid polymer electrolyte. The carbon electrode was fabricated using 90 wt.% of activated carbon and 10 wt.% of poly(vinylidene difluoride) as a binder. Further, the weight ratio (90:10 wt.%) of the working electrode material was mixed well with the help of a mortar and pestle, and a few drops of N-methyl pyrrolidone solvent were added to create a slurry of mixed electrode materials. The slurry was coated onto the nickel foil (current collector area of 1 × 2 cm^2^) substrate using the doctor blade technique. Then, the substrate was dried at 50 °C overnight in a vacuum oven. The activated carbon (AC) electrodes were used to fabricate a symmetric supercapacitor device, and between the electrodes, a PVP/PEO/20 wt.% GuN solid-state-flexible electrolyte (highest ionic conductivity) was used. Thereby, its performance as a separator and solid polymer electrolyte was evaluated. The symmetric supercapacitor device was examined by cyclic voltammetry (CV), galvanostatic charge–discharge (GCD), and electrochemical impedance spectroscopy (EIS).

### 2.4. Characterization Techniques

The crystalline nature of polymer electrolytes was examined by X-ray diffraction. The pattern was recorded using a Bruker D8 advanced X-ray diffractometer instrument (Billerica, MA, USA). To determine the functional group, Fourier transform infrared (FTIR) spectral measurement was carried out using Shimadzu IR Tracer-100 (Kioto, Japan). HIOKI- LCR 3536 (Nagano, Japan) was used for dielectric and impedance studies. The various prepared weight percentages of guanidinium nitrate salt-added PVP/PEO solid polymer electrolytes were used with a frequency range of 4 Hz to 8 MHz. In addition, the thickness of the solid polymer electrodes was 0.7 mm. The surface morphological studies of the polymer electrolytes were studied using Carl Zeiss Model Supra 55 (Oberkochen, Germany). The CHI 660F (Bee Cave, TX, USA) electrochemical system was used to analyze the electrochemical performance of the fabricated device in a two-electrode system.

## 3. Results and Discussion

### 3.1. Structural and Vibrational Studies

**Figure 1:**
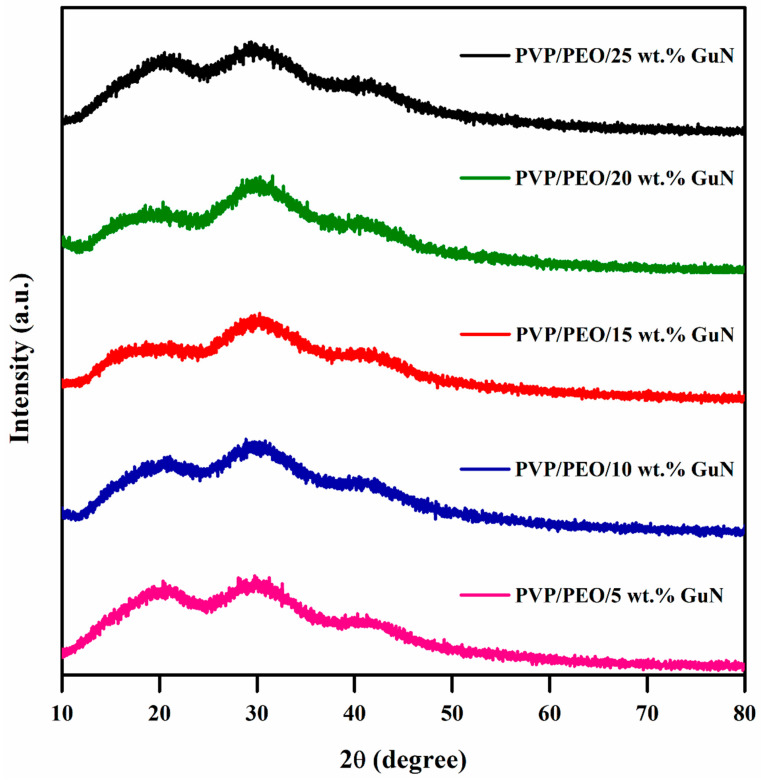
XRD patterns of solid polymer electrolytes.

Figure 1 shows the XRD patterns of different concentrations of GuN with PVP/PEO polymer matrices. The XRD patterns of the prepared PVP/PEO/X wt.% GuN showed broad hubs in 2θ values of 20°, 29°, and 42°. These peaks were caused by strong interactions between molecules on the polyether side chain of the PEO polymer [11,12]. The XRD patterns of pure GuN and the blended polymer PVP/PEO are given in the Supplementary Information (see Figure S1). The use of salt reduced the intensity of the peak while increasing its broadness. This suggests a rise in the amorphous character of polymer electrolytes. There were no more guanidinium salt peaks seen up to 25 wt.% composition, which confirmed that the salt was entirely dissolved in the electrolyte. This indicates the complete dissolution of GuN in the polymer blend matrices. The amorphous nature led to high ionic conductivity through the chain of polymer blend matrices [11].

**Figure 2:**
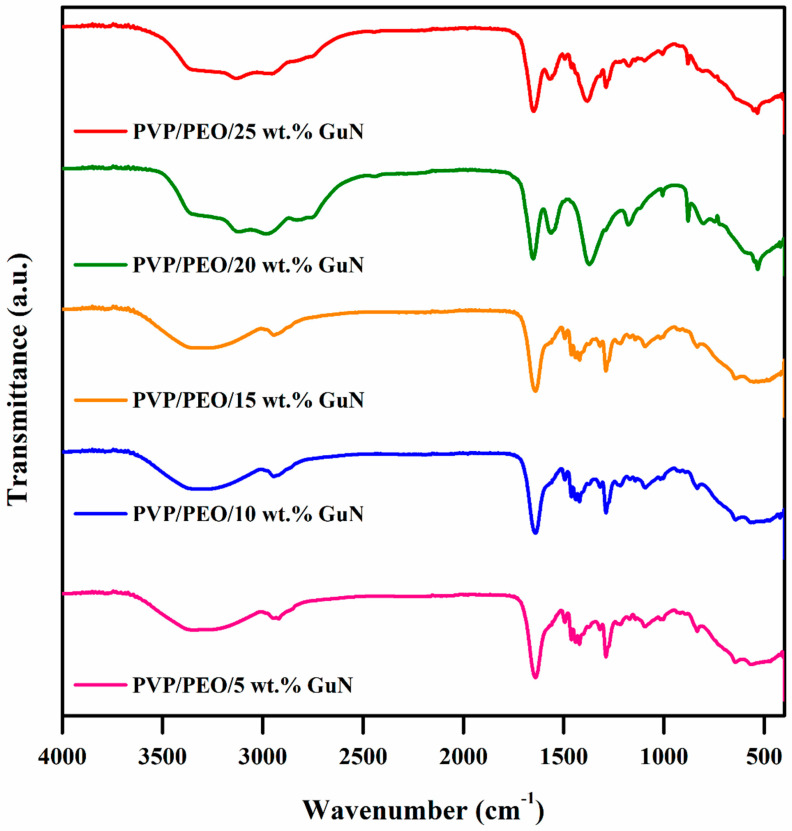
FTIR spectra of PVP/PEO/x wt.% GuN solid polymer electrolytes.

Figure 2 depicts the Fourier transform infrared spectra of GuN-added PVP/PEO electrolytes. This powerful approach revealed intermolecular interactions between PVP and a PEO polymer mix, as well as interactions with guanidinium–OH stretching, CH_2_ scissoring, CH_2_ asymmetric stretching, the C–O–C stretching mode of PEO, symmetric and asymmetric C=O, and C–H asymmetric stretching of the ethylene group. FTIR vibration bands at 2886, 1680, 1463, 1340, 1280, 1096, and 957 cm^−1^ showed C–O stretching with some CH₂ asymmetric rocking and CH₂ rocking in PVP, as well as some C–O stretching in PEO [11,12,21]. Two additional peaks appeared at 3360 (stretching vibration of NH_2_ for guanidinium nitrate salt) and 549 cm^−1^, assigned as the bending mode of NH_2_ [22]. As the GuN ratio rose, the FT-IR spectra were likely to shift due to the interaction of the guanidinium ion with the PEO and PVP polymer matrix. The FT-IR spectra of pure GuN, PVP and PEO are given in the Supplementary Information (see Figure S2). In this case, N-H stretching vibrations from the guanidinium salt may have interacted with the polymer’s hydroxyl groups, causing a shift to lower wavenumbers in the range of 600–700 cm^−1^.

### 3.2. Surface Morphological Analysis

**Figure 3:**
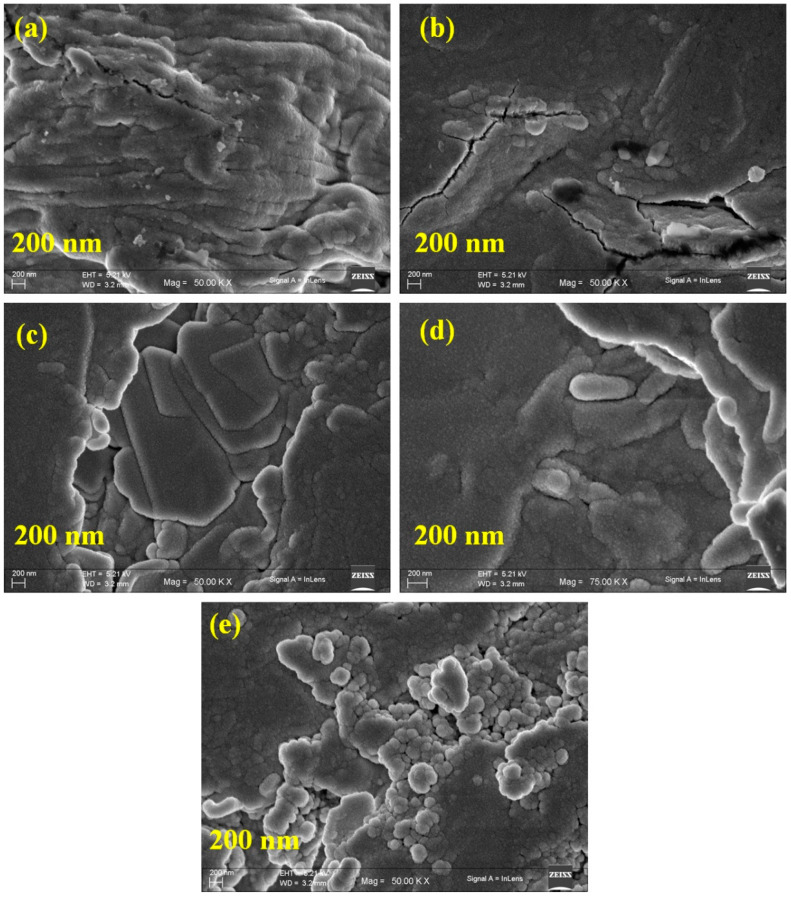
SEM images of PVP/PEO/X wt.% GuN solid polymer electrolyte: (**a**) 5 wt.%, (**b**) 10 wt.%, (**c**) 15 wt.%, (**d**) 20 wt.%, and (**e**) 25 wt.%.

Figure 3 shows a morphological view of the blend polymeric system with different weight percentages of GuN salt. There was no phase separation, indicating homogeneity due to possible molecular-level interactions between PVP and PEO. Figure 3 illustrates the surface morphology of the blended PVP/PEO/X wt.% GuN electrolytes. The distribution of the guanidinium nitrate salt in a PVP/PEO matrix could be observed in the SEM analysis. It showed that the guanidinium nitrate salt was uniformly distributed in the polymer electrolytes with homogeneous surfaces [23]. The prepared polymer electrolyte surface had numerous gaps and voids of different sizes, as seen in the SEM images. After this analysis, the guanidinium salt was fully dissolved and spread over the polymeric system. At higher salt concentrations (25 wt.% of GuN), the surface morphology revealed a roughened surface with larger GuN agglomerates, which could reduce ion conductivity.

### 3.3. Complex Impedance Plot Analysis 

**Figure 4:**
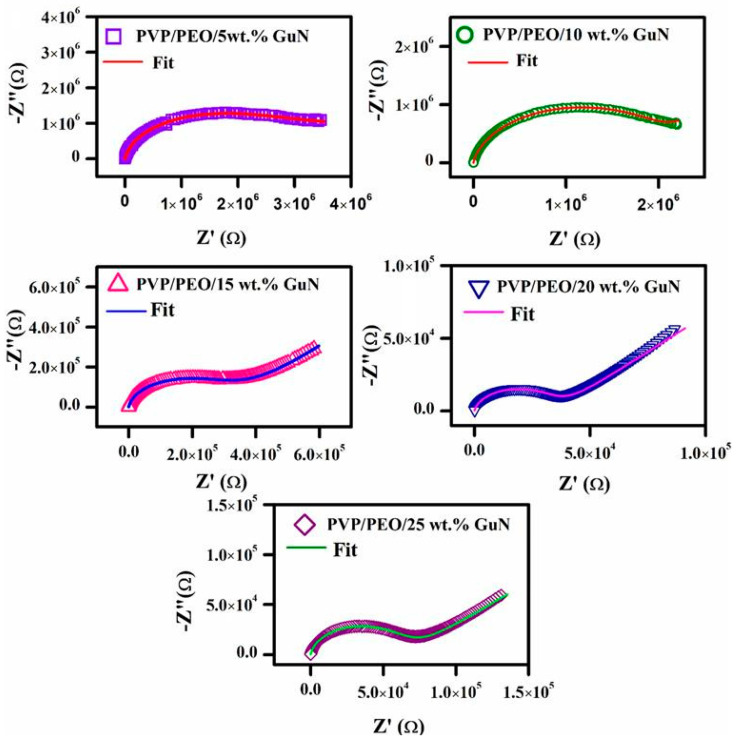
Complex impedance plot of various compositions of PEO/PVP/GuN SPEs.

A Nyquist plot (−Z″ Vs Z′) of various compositions of PVP/PEO/GuN (70/30/5–25 wt.%) SPEs is shown in Figure 4. All composites exhibited single semicircles with oblique spikes at high and low frequencies, respectively. This occurred due to a parallel connection of mobile and immobile charge carriers with constant phase element (CPE) series connections [22]. The spike was observed to be shorter at the initial concentration and longer at the higher salt concentration thereafter. Of these, the 20 wt.% GuN SPE had the longest slanted line. The length of the slant line rose with increasing charge accumulation between the electrode and electrolyte interface. This confirms the capacitance effect, which was highest in the 20 wt.% GuN SPEs [24]. The end of the semicircle tapered down to the 20 wt.% GuN composition and then began to increase. The elongation of the semicircle intersecting the x-axis represents the bulk resistance (R_b_) of the corresponding electrolyte and was measured by fitting a complex impedance plot using *Z-View* 4.0i software (Southern Pines, NC, USA). CPE-T and CPE-P are two parameters of a constant phase element (CPE) that were used to calculate the charge-transfer resistance and other values. The calculated constant phase element values of SPEs are given in Table 1. The conductivity of the SPEs was measured with the following equation, along with the R_b_, thickness (t), and contact area (A).

(1) $$\sigma =\frac{t}{R_{b}A}\;S/cm$$

The calculated conductivity values of different wt.% GuN-based SPEs are listed in Table 2. Up to 20 wt.%, the conductivity value increased (1.84 × 10⁻⁶ S/cm) with increases in mobile charge carriers and then decreased with increases in immobile charge carriers. Due to the high ion dissociation rate, the conductivity value generally increased with the increase in the GuN salt concentration [25,26]. Based on this observation, guanidinium nitrate-added polymer electrolytes exhibited higher conductivity compared with guanidinium carbonate-added PVP/PEO electrolytes [12]. The comparative analysis is given in the Supplementary Information (see Table S1).

### 3.4. Conductance Spectral Study

**Figure 5:**
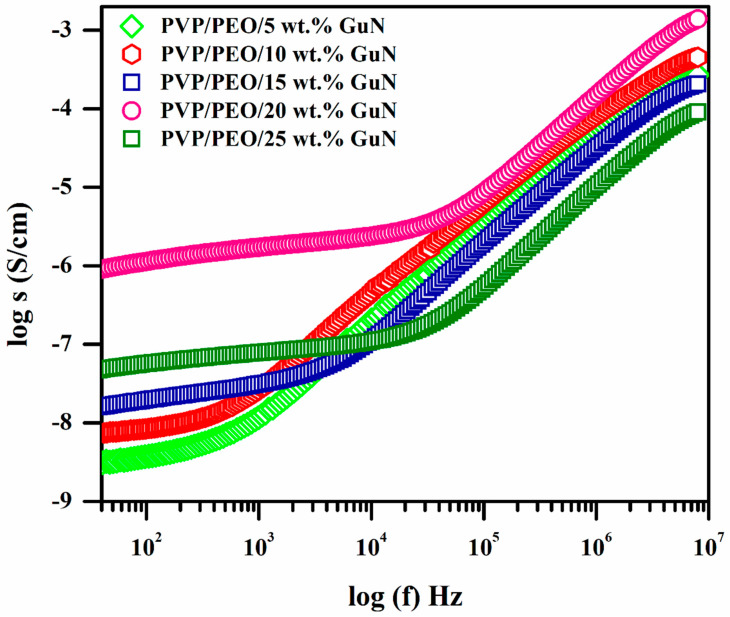
Conductance spectra for different compositions of PEO/PVP/GuN SPEs.

Notable changes in the conductance behavior with frequency at room temperature for various wt.% GuN-added PVP/PEO-based polymer electrolytes are shown in Figure 5. The low-frequency plateau region was responsible for the AC conductivity of the polymer electrolytes. The availability of mobile charge carriers increased due to increases in the GuN concentration in SPEs, which were also the reason for the AC conductivity increment up to 20 wt.% GuN [27]. A lack of mobile charge carriers decreased the AC conductivity of higher GuN concentrations. The length of the plateau region (along x-axis) also increased with increments in GuN concentration in SPEs. At the high-frequency dispersion region, the mobility of the charge carriers was increased so that the AC conductivity was high at a higher frequency rather than a low frequency [28].

### 3.5. Dielectric Studies

**Figure 6:**
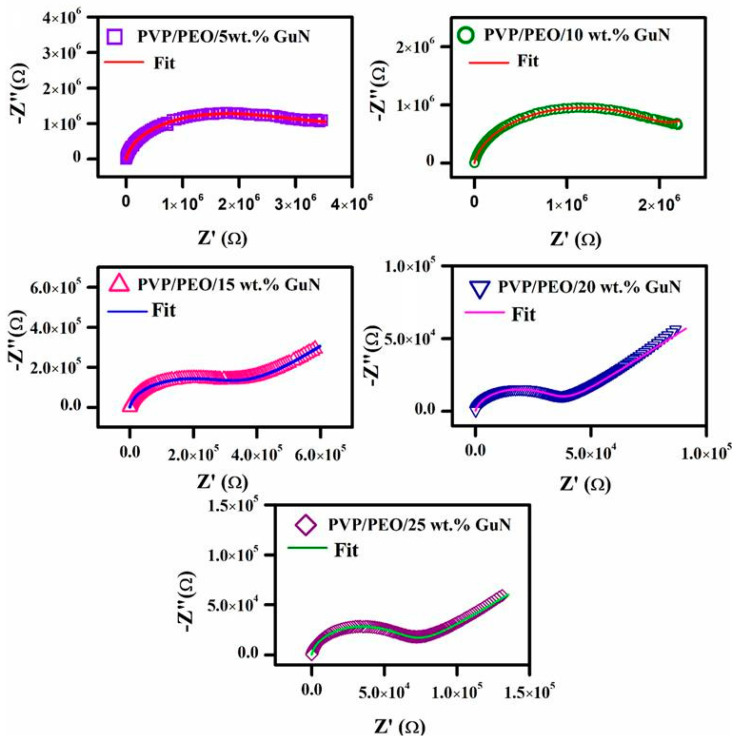
Dielectric constant (solid) and dielectric loss (open) versus frequency for different compositions of PEO/PVP/GuN SPEs.

Dielectric properties analysis is essential for SPEs during use in energy storage applications. The dielectric constant (ε′) and dielectric loss (ε″) with the frequency of the PVP/PEO/GuN-based SPEs at ambient temperature are shown in Figure 6. Although the values of both ε′ and ε″ were initially high at a low frequency due to space charge polarization or electrode–electrolyte interface effect [29], their values decreased and reached a steady state as they moved towards higher frequencies. The reason for the steady state is that there was insufficient time for ion orientation at a high frequency in the applied electric field [30].

There was no change in ε′ or ε″ at high frequency, and the values of the dielectric constant and dielectric loss increased up to 20 wt.% GuN due to the increase in the mobile charge carrier with the increasing salt concentration. Also, the difference between the values of ε′ and ε″ in this composition was very small compared to others. Above this concentration, its values decreased due to reduced mobile charge carrier availability due to high charge accumulation. This result reconfirms that the 20 wt.% GuN had the best performance.

### 3.6. Dielectric Modulus Analysis

**Figure 7:**
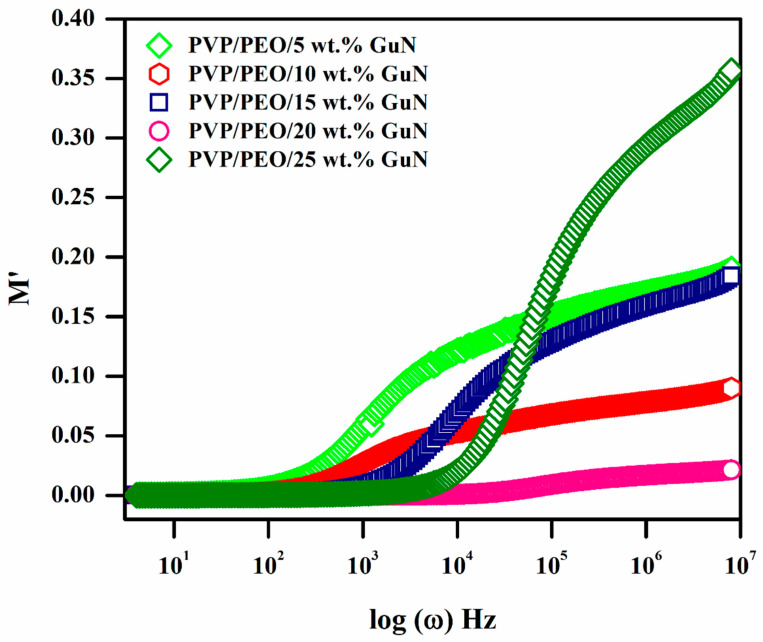
Variation in real modulus with frequencies of different compositions of PEO/PVP/X wt.% GuN SPEs.

The variation in the real modulus (M′) of the SPEs with frequency can be seen from Figure 7. The relation between complex permittivity (ε*) and complex modulus (M*) can be understood by using the following equation [31].

(2) $$M^{*}=\frac{1}{\varepsilon^{*}}=M^{\prime }+jM^{\prime \prime }=\frac{\varepsilon^{\prime }}{{\varepsilon^{\prime }}^{2}+{\varepsilon^{\prime \prime }}^{2}}+j\frac{\varepsilon^{\prime \prime }}{{\varepsilon^{\prime }}^{2}+{\varepsilon^{\prime \prime }}^{2}}$$

The observation of a long tail of the zero level at a low frequency confirms the absence of the contribution of space charge polarization. The length of this tail also increased as the presence of GuN concentration increased. Among these, the 20 wt. % GuN electrolyte had the longest tail, indicating the best capacitive effect.

**Figure 8:**
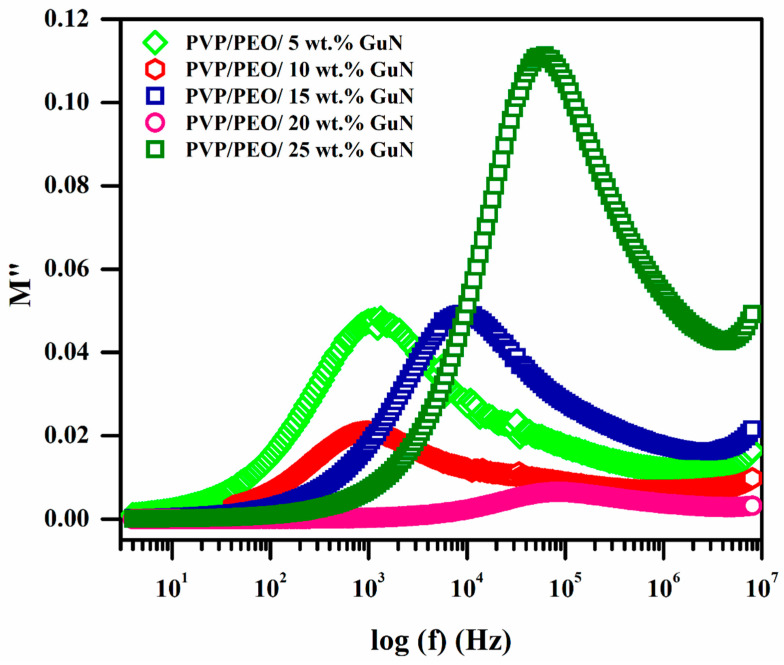
Variation in imaginary modulus with frequencies of different compositions of PEO/PVP/GuN SPEs.

The values of M’ increased at higher frequencies and reached the saturation level due to the relaxation process. Figure 8 shows the variation in the imaginary modulus (M″) with the frequencies of different wt.% GuN-added PVP/PEO-based SPEs. Upon increasing the frequency, initially, the values of M″ increased until reaching a maximum and then decreased. The below -M″_max_ region shows this due to the long-range order of mobile charge carriers, whereas the beyond -M″_max_ region shows that the mobile charge carriers were bound in their potential wells [32]. With the increasing GuN concentration, M″_max_ shifted towards a higher frequency, indicating that it reached a minimum relaxation time, and the observed asymmetric peaks confirm its non-Debye nature [33].

### 3.7. Kohlrausch–Williams–Watts (KWW) Parameter Analysis

A proper fitting of the M″ plot with the Bergma-proposed modified KWW function provides the details of M″_max_, f_max_, stretching exponent parameter (β), and relaxation time (τ). The relaxation function of the SPEs can be described by the following modified KWW function [34].

(3) $$M^{\prime \prime }=\frac{M_{max}^{\prime \prime }}{\left(1-\beta \right)+\frac{\beta }{1+\beta }\left[\beta \left(\frac{f_{max}}{f}\right)+{\left(\frac{f}{f_{max}}\right)}^{\beta }\right]}$$

**Figure 9:**
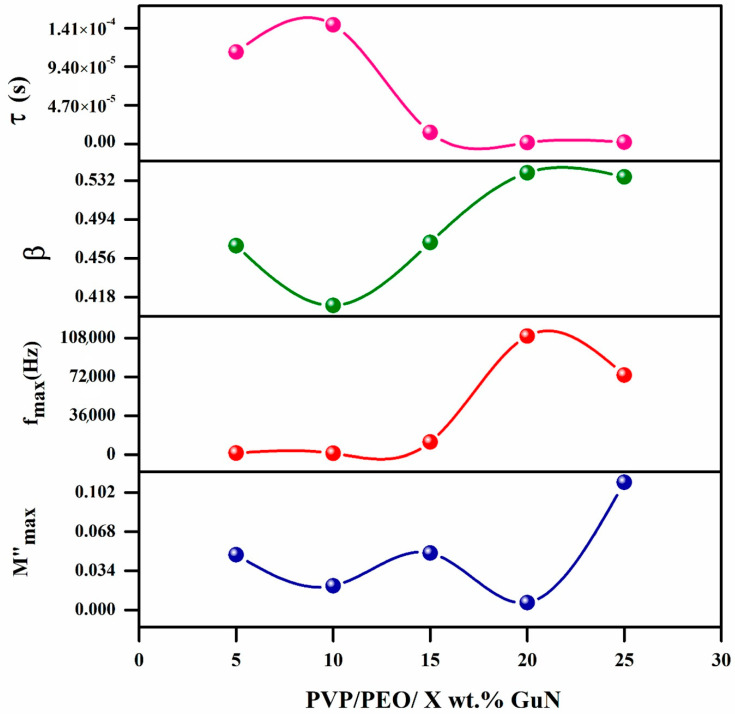
M″_max_, f_max_, stretching exponent parameter, and relaxation time versus GuN concentration.

The measured KWW parameters of M″_max_, f_max_, β, and τ are listed in Table 3. The M″_max_ versus GuN concentration plot (Figure 9) shows irregular changes. Among these, 20 wt.% GuN was also found to have the lowest M″_max_ value, as the variation in the dielectric constant and dielectric loss was very low. Figure 9 shows the variation in f_max_ with different wt.% GuN concentrations, since f_max_ is the corresponding frequency of M″_max_. f_max_ moved towards a higher frequency up to 20 wt.% GuN and then decreased. so that 20 wt.% GuN composition would reach maximum values. The variation in β values with GuN concentration is revealed in Figure 9. The value of β was always between 0 and 1. The β value is unified for an ideal Debye relaxation type. But here, the β value is below 1, so the relaxation deviates greatly from the ideal Debye type, confirming that these materials follow the non-Debye type of relaxation [35,36,37]. The inverse of frequency at corresponding maximum peak position gives the relaxation time (τ = 1/(2π f_max_)). It is evident from Figure 9 that the 20 wt.% solid polymer electrolyte achieved the lowest relaxation time of 1.45 μs due to this composition containing more mobile charge carriers than the others. The presence of free ions, combined with reduced ion pairing, allowed for faster ionic mobility within the polymer matrix, contributing to a decrease in the relaxation time. Ions respond rapidly to external stimuli like electric fields, resulting in reduced relaxation times.

### 3.8. Electrochemical Performance of Flexible Solid Polymer Electrolyte

**Figure 10:**
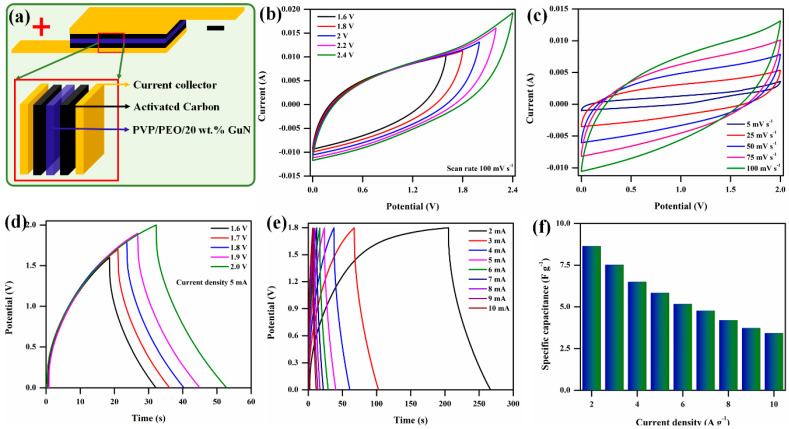
(**a**) Schematic view of symmetric supercapacitor device, (**b**,**c**) CV profile, (**d**,**e**) GCD curves, and (**f**) specific capacitance and current density.

The electrochemical properties of the fabricated symmetric supercapacitor device were evaluated by CV in a PVP/PEO/20 wt.% GuN solid polymer electrolyte. A schematic illustration of a symmetric supercapacitor device is given in Figure 10a. Figure 10b,c shows the CV curves of the fabricated symmetric supercapacitor device with different window potentials of 1.6 V to 2.4 V (interval of 0.2 V) and a similar scan rate of 100 mV s⁻¹. While increasing the scan rate, the rectangular shape of the voltammogram for the symmetric supercapacitor device was maintained up to 100 mV s^−1^, which shows the good rate capability of the device [38]. Non-faradic reactions involve rectangular shapes in CV curves, where charge storage occurs via adsorption at the interface of electrolytes and electrode surfaces. This confirms the ideal electrical double-layer capacitive (EDLC) behavior of the symmetric supercapacitor device [39] and depends only on electrolyte ions that are intercalated into the electrode surface [40].

Furthermore, the symmetric supercapacitor device was studied via galvanostatic charge–discharge at different window potentials of 1.6 to 2 V with a current density of 5 A g^−1^. In Figure 10d,e, the 1.8 V potential shows nearly symmetrical triangles. Thus, the potential window was fixed to allow for study at different current densities, such as 2 to 10 A g^−1^, which is characteristic of an EDLC capacitor with good electrochemical reversibility [11,15]. The specific capacitance (C_sp_) of the fabricated device was calculated by the following equations.

(4) $$C_{sp}=\frac{I\times ∆t}{∆V\times m}\;{F\;g}^{-1}$$

(5) $$\eta =\frac{t_{d}}{t_{c}}\times 100\;(\%)\;$$

**Figure 11:**
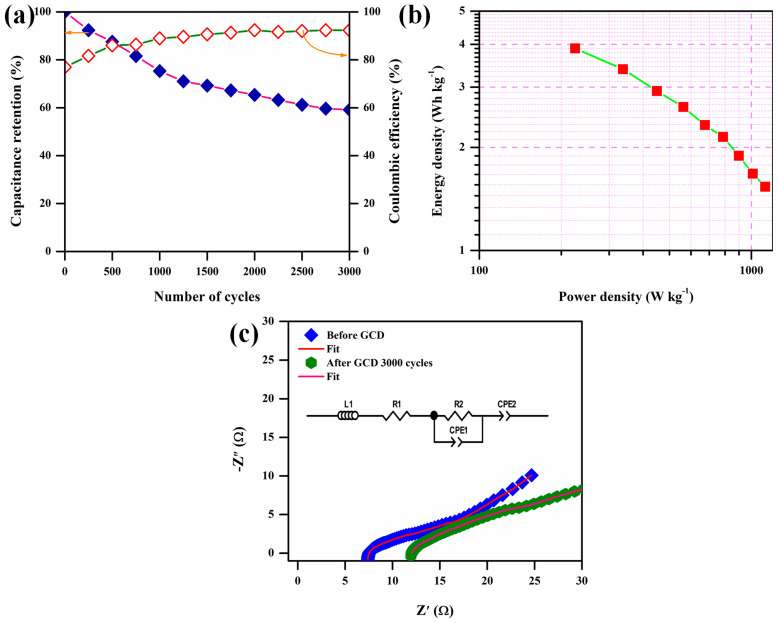
(**a**) Capacitance retention, (**b**) Ragone plot, and (**c**) EIS of fabricated symmetric supercapacitor device.

The specific capacitance of the symmetric supercapacitor device was calculated and is shown in Figure 10f. Due to the lower current density, the device took a longer time to discharge due to sufficient time for the electrolyte ions to diffuse off the activated carbon electrode, demonstrating the higher charge storage capacity of the symmetric supercapacitor device [41]. The specific capacitance was decreased from 8.64 F g^−1^ to 3.41 F g^−1^ as the current density increased from 2.0 A g^−1^ to 10 A g^−1^, respectively. Because there was enough time for the electrolyte ions to adsorb and desorb on the electrode surface, the data demonstrate that the specific capacitance dropped as the current density increased. The long-term cycle stabilities of the fabricated symmetric supercapacitor devices were examined up to 3000 GCD cycles at a current density of 5 A g^−1^. The symmetric supercapacitor device exhibited a cycle life of 59.16% over 3000 cycles and is shown in Figure 11a. The Coulombic efficiency of the symmetric supercapacitor was calculated using Equation (5) and a calculated Coulombic efficiency of 92.36 % after GCD 3000 cycles. The energy and power densities of the fabricated symmetric supercapacitor device was calculated from GCD at different current densities. The energy (E) and power density (P) were calculated by the following equations.

(6) $$E=\frac{0.5\times C_{sp}\times ∆V^{2}}{3.6}\;Wh\;{kg}^{-1}$$

(7) $$P=\frac{E\times 3600}{∆t}\;W\;{kg}^{-1}$$

Figure 11b shows the Ragone plot of the fabricated symmetric supercapacitor device. The superior E was observed with 3.88 W h kg^−1^ and a power density of 1132 W kg^−1^. The efficiency of the PVP/PEO/20 wt.% GuN-based solid polymer electrolyte as an efficient electrolyte material for supercapacitors is supported by its superior energy density [40]. EIS was carried out for the fabricated symmetric supercapacitor device [42,43], and the EIS plots are shown in Figure 11c. The EIS was examined with a frequency region of 1 Hz to 100 kHz and an applied potential amplitude of 5 mV. The EIS plot describes the intrinsic properties of electrode materials in three major segments, i.e., the high-, middle-, and low-frequency regions. We determined the series resistance (Rs) and charge transfer resistance (R_ct_) before and after 3000 GCD cycles with the help of *Z-View* fit software. The diffusion of ions on the electrode surface was responsible for the low-frequency region, which is indicative of capacitive behavior [44,45,46]. The Rs values were 7.519 Ω and 11.996 Ω before and after 3000 GCD cycles, respectively. The calculated R_ct_ values were 3.319 Ω and 6.353 Ω before and after the GCD cycles, respectively. The R_s_ and R_ct_ values increased after 3000 GCD cycles due to continue charge–discharge, distortion of the electrode porous structure, a decrease in the area of the active site of the electrode, and the lower number of mobile ions that were accessible for charge transfer [45,46].

## 4. Conclusions

The hydrogen-rich guanidinium nitrate-added PVP/PEO-based solid polymer electrolytes were prepared by a simple solution casting method at room temperature. According to X-ray diffraction analysis, the crystalline peak for PEO was reduced with the increasing salt concentration, suggesting a shift toward its amorphous nature. The FTIR spectra demonstrated that when the salt concentration increased, the unique bands of PEO and PVP polymer matrices tended to shift. Up to 20 wt.% GuN, the conductivity value increased (1.84 × 10⁻^6^ S/cm) with an increase in mobile charge carriers. With the increasing GuN concentration, M_max_ shifted towards a higher frequency, indicating that it reached a minimum relaxation time. The electrochemical properties of the fabricated symmetric supercapacitor device of 20 wt.% GuN-added PVP/PEO were evaluated, and at a lower current density, the device took a longer time to discharge due to sufficient time for the electrolyte ions to diffuse from the activated carbon electrode. Furthermore, it exhibited outstanding cycling stability for up to 3000 galvanostatic charge–discharge cycles, and 59.16% retention was obtained. These results demonstrate the potential applications of solid polymer electrolytes in the progression of flexible supercapacitor applications.

## Figures and Tables

**Table 1:** Calculated constant phase element values of SPEs.

Elements	PVP/PEO/5 wt.% GuN	PVP/PEO/10 wt.% GuN	PVP/PEO/15 wt.% GuN	PVP/PEO/20 wt.% GuN	PVP/PEO/25 wt.% GuN
CPE-T (Ω cm^2^)	1.078 × 10^−8^	1.868 × 10^−8^	6.512 × 10^−7^	2.517 × 10^−6^	2.936 × 10^−6^
CPE-P (F s(n^−1^))	0.5311	0.3064	0.2791	0.4796	0.4014

**Table 2:** Conductivity values for different wt.% GuN with PVP/PEO-based SPEs.

Electrolyte	Conductivity σ_ac_ (S/cm)
PVP/PEO/5 wt.% GuN	5.81 × 10^−10^
PVP/PEO/10 wt.% GuN	2.14 × 10^−9^
PVP/PEO/15 wt.% GuN	1.22 × 10^−8^
PVP/PEO/20 wt.% GuN	1.84 × 10^-6^
PVP/PEO/25 wt.% GuN	4.03 × 10^−8^

**Table 3:** KWW parameters for polymer electrolytes.

Electrolyte	M″_max_	f_max_ (Hz)	β	τ (s)
PVP/PEO/5 wt.% GuN	0.048	1423.72	0.468	1.12 × 10^−4^
PVP/PEO/10 wt.% GuN	0.021	1100.40	0.410	1.45 × 10^−4^
PVP/PEO/15 wt.% GuN	0.0494	11,470.78	0.471	1.39 × 10^−5^
PVP/PEO/20 wt.% GuN	0.0064	109,733.13	0.540	1.45 × 10^−6^
PVP/PEO/25 wt.% GuN	0.111	73,609.86	0.535	2.16 × 10^−6^

## Data Availability

The data presented in this study are available on request from the corresponding author.

## Supplementary Materials

The following supporting information can be downloaded at: https://www.mdpi.com/article/10.3390/polym17020206/s1, Figure S1: PXRD patterns of PVP/PEO and GuN; Figure S2: FTIR spectra of pure GuN, PVP and PEO. Table S1: Conductivity analysis of guanidinium salt added PVP/PEO matrix.
