# Supplementary material for: Electrochemical Performance of Guanidinium Salt-Added PVP/PEO Solid Polymer Electrolyte with Superior Power Density
Source: Polymers (Basel). 2025 Jan 15;17(2):206. doi: 10.3390/polym17020206 (PMC11769341; doi:10.3390/polym17020206)
Supplement: Supplementary file 1 [file polymers-17-00206-s001.zip › polymers-3306962-supplementary.pdf]

Supplementary Materials: Electrochemical Performance of Guanidinium Salt-added PVP/PEO Solid Polymer Electrolyte with Superior Power Density

Anbazhagan Murugan [1,2], Vadivel Siva [2,*], Abdul Samad Shameem [3], Paranthaman Vijayakumar [4], Arangarajan Viji [5], Jintae Lee [6] and Govindasamy Palanisamy [6,*]

[1] Department of Science and Humanities, Karpagam College of Engineering, Coimbatore 641032, Tamil Nadu, India
[2] Department of Physics, Centre for Energy and Environment, Karpagam Academy of Higher Education, Coimbatore 641021, Tamil Nadu, India
[3] Department of Science and Humanities, Karpagam Academy of Higher Education, Coimbatore 641021, Tamil Nadu, India
[4] Department of Electrochemistry, Saveetha School of Engineering, SIMATS, Chennai 602105, Tamil Nadu, India; vijayakumar@uniten.edu.my
[5] Department of Physics, Kongunadu College of Engineering and Technology, Thottiyam, Tiruchirappalli 621215, Tamil Nadu, India
[6] School of Chemical Engineering, Yeungnam University, 280 Daehak-ro, Gyeongsan 38541, Republic of Korea
* Correspondence: siva.vadivel@kahedu.edu.in (V.S.); palanisamyg@yu.ac.kr (G.P.)

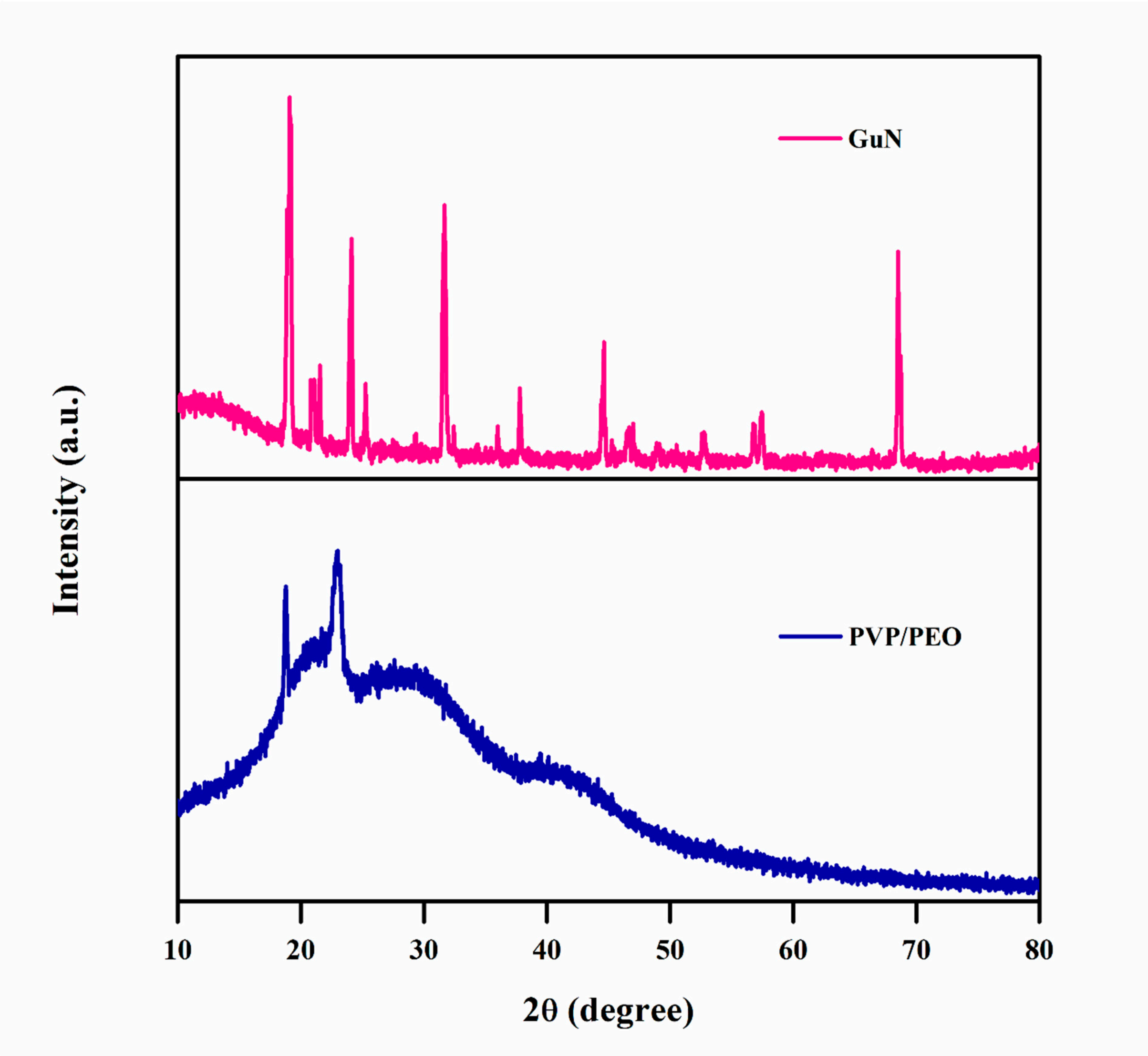

Figure S1. PXRD patterns of PVP/PEO and GuN.

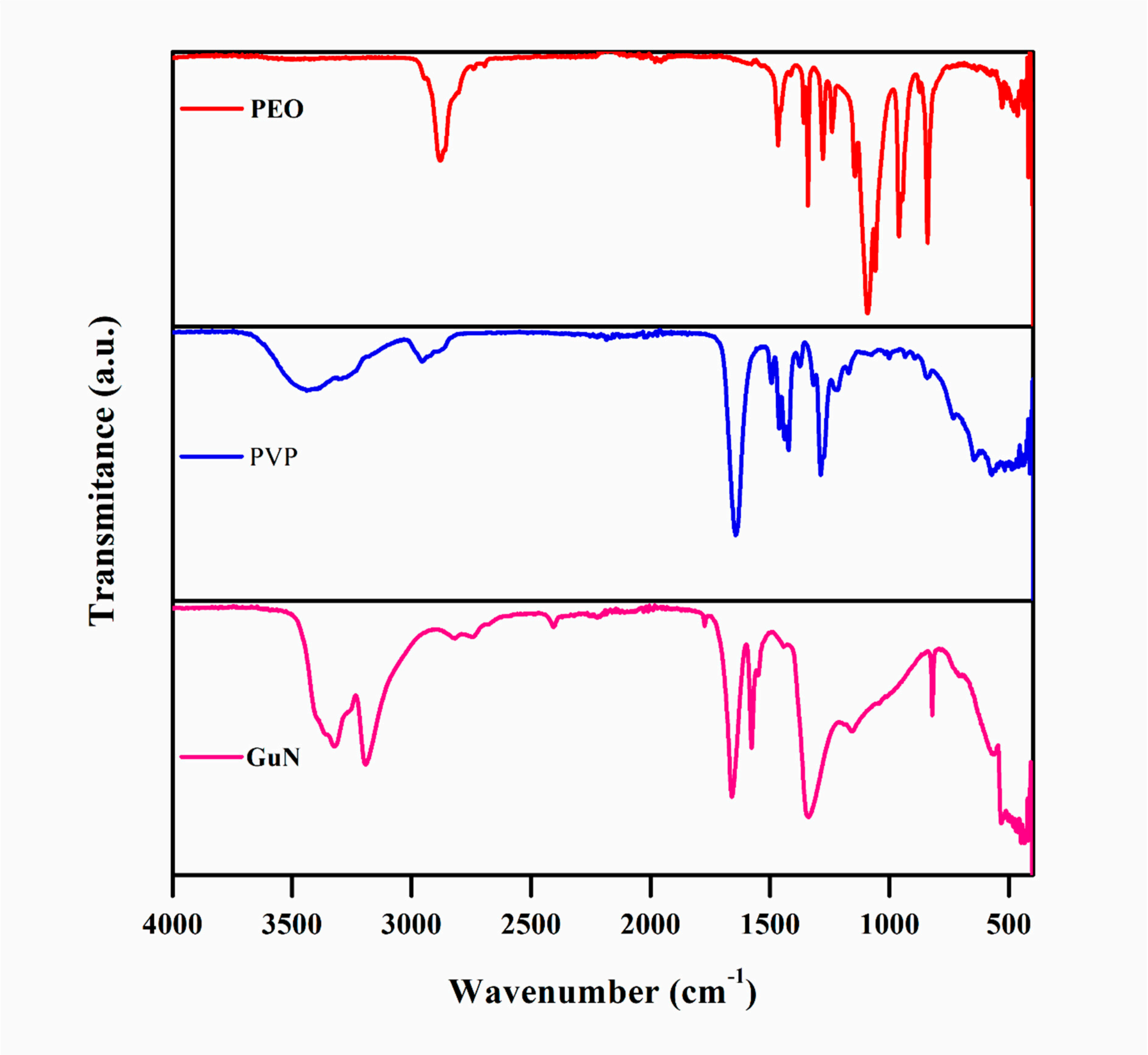

Figure S2. FTIR spectra of pure GuN, PVP and PEO.

Table S1. Conductivity analysis of guanidinium salt added PVP/PEO matrix.

**PVP/PEO/Guanidinium carbonate [12]**		**PVP/PEO/Guanidinium Nitrate**	
**Electrolyte**	**Conductivity (S/cm)**	**Electrolyte**	**Conductivity (S/cm)**
PVP/ PEO/ 5 wt.% GuC	1.80513×10^{-7}	PVP/ PEO/ 5 wt.% GuN	5.81×10^{-10}
PVP/ PEO/ 10 wt.% GuC	5.73162×10^{-8}	PVP/ PEO/ 10 wt.% GuN	2.14×10^{-9}
PVP/ PEO/ 15 wt.% GuC	9.29196×10^{-9}	PVP/ PEO/ 15 wt.% GuN	1.22×10^{-8}
PVP/ PEO/ 20 wt.% GuC	1.50165×10^{-8}	**PVP/ PEO/ 20 wt.% GuN**	**1.84×10^{-6}**
PVP/ PEO/ 25 wt.% GuC	3.00764×10^{-7}	PVP/ PEO/ 25 wt.% GuN	4.03×10^{-8}
